# Exploring the design of clinical research studies on the efficacy mechanisms in type 2 diabetes mellitus

**DOI:** 10.3389/fendo.2024.1363877

**Published:** 2024-09-20

**Authors:** Huifang Guan, Shuang Zhao, Jiarui Li, Ying Wang, Ping Niu, Yuxin Zhang, Yanjiao Zhang, Xinyi Fang, Runyu Miao, Jiaxing Tian

**Affiliations:** ^1^ College of Traditional Chinese Medicine, Changchun University of Chinese Medicine, Changchun, China; ^2^ Department of Encephalopathy, The Affiliated Hospital of Changchun university of Chinese Medicine, Jilin, China; ^3^ Institute of Metabolic Diseases, Guang’anmen Hospital, China Academy of Chinese Medical Sciences, Beijing, China; ^4^ Graduate College, Beijing University of Chinese Medicine, Beijing, China

**Keywords:** type 2 diabetes mellitus (T2DM), omics technologies, personalized treatment, metabolomics, glycolipid metabolism

## Abstract

This review examines the complexities of Type 2 Diabetes Mellitus (T2DM), focusing on the critical role of integrating omics technologies with traditional experimental methods. It underscores the advancements in understanding the genetic diversity of T2DM and emphasizes the evolution towards personalized treatment modalities. The paper analyzes a variety of omics approaches, including genomics, methylation, transcriptomics, proteomics, metabolomics, and intestinal microbiomics, delineating their substantial contributions to deciphering the multifaceted mechanisms underlying T2DM. Furthermore, the review highlights the indispensable role of non-omics experimental techniques in comprehending and managing T2DM, advocating for their integration in the development of tailored medicine and precision treatment strategies. By identifying existing research gaps and suggesting future research trajectories, the review underscores the necessity for a comprehensive, multidisciplinary approach. This approach synergistically combines clinical insights with cutting-edge biotechnologies, aiming to refine the management and therapeutic interventions of T2DM, and ultimately enhancing patient outcomes. This synthesis of knowledge and methodologies paves the way for innovative advancements in T2DM research, fostering a deeper understanding and more effective treatment of this complex condition.

## 1 Introduction

In recent years, research in Type 2 Diabetes Mellitus (T2DM) within the realm of glycolipid metabolism has achieved notable progress, uncovering the intricate links between the disorder, its complications, and glycolipid metabolic dysregulation (1). These discoveries have not only shed light on the biological underpinnings of the disease but also provided crucial biomarkers and therapeutic targets (2, 3). Particularly noteworthy is the shift in the focus of clinical research on pharmacodynamics from single-target drug effects to multi-target interventions and personalized treatment strategies (4). This transition reflects a profound understanding of the complex pathophysiological mechanisms of diabetes and acknowledges the individual variances and disease heterogeneity among patients. Understanding the individual variability in response to treatments is crucial for the effective management of T2DM. This variability can be attributed to several factors, including genetic diversity, epigenetic modifications, differences in protein expression, metabolic profiles, and the composition of the gut microbiome.

Against this backdrop, the application of omics technologies and basic experimental methods has opened new avenues for exploring the glycolipid metabolic mechanisms in T2DM, thereby offering more precise guidance for novel drug development and clinical treatments. Omics technologies have revolutionized our understanding of T2DM, tracing a trajectory from foundational genomic associations to sophisticated multi-omic integrations. Initially, genome-wide association studies unveiled genetic susceptibilities crucial for T2DM, laying the groundwork for personalized genetic risk profiling (5, 6). Advancements in transcriptomics have provided intricate details on cellular diversities that affect insulin production and action, thus enhancing our understanding of beta-cell dysfunction (7). Concurrently, advancements in proteomics have demonstrated its potential in early detection and intervention in T2DM, as evidenced by a study using the urinary proteomic classifier CKD273, which predicted the risk of progression to microalbuminuria and informed therapeutic decisions in diabetic kidney disease (8). Further expanding our perspective, metabolomics has identified metabolic precursors that herald symptomatic diabetes, offering targets for early intervention (9). Complementarily, microbiomics has revealed the crucial role of gut microbiota in regulating systemic metabolic health, significantly influencing glucose tolerance (10). This comprehensive narrative across omics fields highlights a paradigm shift from mere glucose management to an integrated view of metabolic interplay, thereby paving the way for precise therapeutic interventions tailored to individual metabolic profiles.

While omics technologies have profoundly enhanced our understanding of T2DM, current methodologies still face significant limitations, particularly in biomarker monitoring within clinical samples and in the detailed mechanistic and pharmacodynamic studies these biomarkers inform. In clinical practice, we frequently collect samples from a variety of patient groups, including those in the early, middle, and late stages of disease, as well as from different treatment cohorts. Traditionally, the use of these clinical samples in research has been underutilized. However, recent advancements in omics technologies have revolutionized our ability to conduct deeper analyses of these samples, thereby enhancing our capacity to precisely assess their relevance to specific clinical questions. This paradigm shift represents a novel research strategy that significantly improves our understanding of Type 2 Diabetes Mellitus (T2DM) by integrating clinical observations with molecular insights. By concentrating on studies that utilize clinical samples, our review aims to provide researchers with strategic guidance on the direction of T2DM studies and the categorization of samples, informed by the latest developments in omics technologies.

Addressing these limitations, the primary aim of this paper is to explore several crucial questions: How can clinical research samples be selected efficiently and scientifically? Which cutting-edge biotechnological methods should be employed to ensure the accuracy and depth of the data during sample processing? How can these methods be utilized to delve deeply into the mechanistic pathways within the samples? Once preliminary findings are obtained from these analyses, how should basic experiments be designed to validate and further investigate these findings?

This review delves into the study design for clinical research on the efficacy mechanisms in T2DM, with a focus on how the integration of advanced omics technologies and traditional basic experimental methods can unravel the complexity of T2DM. We will comprehensively discuss the application of various omics approaches, including genomics, methylation, transcriptomics, proteomics, metabolomics, and intestinal microbiomics, in understanding the mechanisms of T2DM. These methodologies provide profound insights into T2DM from multiple biological perspectives, thereby broadening our comprehensive understanding of the disease’s progression. Additionally, this paper will also focus on non-omics basic experimental techniques, such as cell culture, animal model studies, and molecular biology experiments, which are equally vital for understanding and treating T2DM. These multidimensional research methods pave new pathways for the advancement of personalized medicine and precision treatments, and offer innovative study design approaches for the future treatment and management of diabetes.

## 2 Research methodology

In this review, we implemented a systematic and phased literature search strategy, spanning publications from January 1, 2022, to the present, to ensure the timeliness and innovativeness of our research findings. Our search was concentrated on all major biomedical databases including PubMed, Scopus, Web of Science, and Google Scholar. These databases were chosen for their extensive and diverse resources in biomedical and clinical research. We primarily included clinical trials and a select number of meta-analyses related to genomic GWAS data, explicitly excluding reviews and non-empirical studies to maintain a focus on original research.

To comprehensively explore the application of omics technologies and basic experimental methods in T2DM, and to observe changes in biomarkers and metabolic alterations due to pharmacological interventions, we employed a detailed, staged search strategy in the PubMed database. Initially, our keywords encompassed “(Type 2 Diabetes Mellitus OR T2DM) AND (non-pharmacological interventions OR lifestyle changes) AND (genomics OR methylation OR transcriptomics OR proteomics OR metabolomics OR intestinal microbiomics OR biochemical analysis OR cytological assessment OR histopathological examination OR clinical marker monitoring) AND (human samples OR clinical studies)” to explore changes in omics and non-omics biomarkers in T2DM under non-pharmacological interventions. Subsequently, we used keywords such as “(Type 2 Diabetes Mellitus OR T2DM) AND (pharmacotherapy OR pharmacological interventions) AND (genomics OR methylation OR transcriptomics OR proteomics OR metabolomics OR intestinal microbiomics OR biochemical analysis OR cytological assessment OR histopathological examination OR clinical marker monitoring) AND (human samples OR clinical studies)” to study the changes in omics and non-omics biomarkers under pharmacological interventions. This search strategy not only encompassed a range of omics methods from genomics, methylation, transcriptomics, proteomics to metabolomics and intestinal microbiomics but also included non-omics methods like biochemical analysis, cytological assessment, histopathological examination, and monitoring of clinical indicators. Through this comprehensive approach, we were able to analyze integratively the biological and clinical changes in T2DM under various intervention measures, providing a solid foundation for understanding its complex pathophysiology and evaluating treatment efficacy.

Furthermore, we established explicit inclusion and exclusion criteria to ensure that our review focused exclusively on original research based on human clinical samples, which are central to our investigation of T2DM. We included only English peer-reviewed articles that reported on studies involving clinical samples directly related to human diseases, excluding non-peer-reviewed articles, case reports, and reviews that did not align with our research theme. The initial search returned 141 articles, which we carefully screened to prioritize those that utilized clinical samples—such as peripheral blood, tissue biopsies, or other patient-derived specimens—in omics analyses. These samples are the cornerstone of our research, as they provide direct insights into the biological mechanisms underlying T2DM in real-world clinical settings. Key information, including study design, sample size, primary findings, and the specific application of omics techniques, was extracted from each selected article. We employed content analysis methods to integrate and interpret the results across these studies, ensuring a coherent narrative that reflects the latest clinical applications of omics technologies. To further enhance the rigor of our review, we used standardized assessment tools, including the Preferred Reporting Items for Systematic Reviews and Meta-Analyses guidelines. These tools helped us evaluate the quality and completeness of the literature reporting, ensuring that our review is both thorough and accurate. Ultimately, 102 articles were selected based on their relevance to clinical samples and their potential to inform new research directions, particularly for clinicians working directly with patient samples. Through this approach, we aim to guide researchers in choosing study directions and categorizing clinical samples based on the latest advancements in omics research.

## 3 Sample selection criteria for clinical research studies in type 2 diabetes mellitus

Designing clinical research studies for T2DM necessitates meticulous planning to ensure the inclusion of diverse patient characteristics, thereby guaranteeing robust and generalizable results (11, 12). T2DM presents a spectrum of metabolic profiles from pre-diabetes to advanced stages, making it crucial to stratify patients by disease stage. This stratification is essential as the predictive power of biomarkers and the efficacy of interventions can vary significantly across different stages of T2DM. Including patients with common comorbidities, such as non-alcoholic fatty liver disease (NAFLD) and T2DM renal complications, is equally important (13–15). These conditions can significantly alter metabolic and genetic markers, potentially skewing the interpretation of omics data.

First and foremost, researchers must precisely and consistently define the phenotype of the study population (16). Misclassifying participants can lead to phenotypic heterogeneity, which decreases both sensitivity and statistical power. Clear phenotype definitions enhance biological homogeneity, thereby increasing statistical power. Demographic diversity enhances the generalizability of the study (17, 18). This approach not only aids in discovering more comprehensive biomarkers but also tailors treatment strategies more effectively in clinical practice, advancing precision medicine in T2DM (19).

Moreover, Sample size is essential to good study design. Calculating the sample size shows the study’s power by considering individual measurement variance, acceptable false-positive rates, and the desired discriminatory power of the platform used. Researchers are encouraged to maximize sample sizes in omics studies (20). Despite significant results from omics studies over the past decade, many show high variability and low reproducibility. There is a need for better documentation and consistency in the types of data collected. Standards for minimum information in omics experiments, such as MIAME and MIAPE, have been established to ensure result interpretability (21). These standards emphasize the importance of thorough data documentation and public availability to enhance reproducibility and transparency in research. Clinical trials are often criticized for their lack of generalizability (22). *A priori* generalizability is based on eligibility criteria to ensure the study population mirrors the target population, while a posteriori generalizability compares enrolled patients to real-world populations. Research suggests *a priori* generalizability can predict a posteriori outcomes, indicating well-defined criteria enhance trial applicability. For example, a study on T2DM trials found that populations selected by eligibility criteria closely resembled real-world patients (22). Focusing on criteria such as age, HbA1c levels, and BMI helps define target, study, and enrolled populations. Databases like OneFlorida Data Trust and CALIBER provide extensive US and UK patient data, allowing for thorough validation and robustness of research findings.

In conclusion, a well-defined, diverse study population and consideration of disease stage, comorbidities, demographic diversity, and genetic backgrounds are essential in T2DM research. These strategies ensure the generation of reliable and applicable results, contributing to accelerate knowledge discovery from omics data by optimal experimental design in T2DM. Here is a proposed table for designing basic experiments to validate omics findings inT2DM research **Table 1**. To complement these strategies, it is also crucial to carefully consider the selection, handling, and preservation of biological samples, as outlined in **Table 2**, which details considerations for various 'omics' platforms in T2DM research.

**Table 1:** Key aspects and details in designing clinical research studies on efficacy mechanisms in type 2 diabetes mellitus.

Aspect	Details
Set the Study Hypothesis	Formulate hypotheses based on omics data (e.g., genomic, transcriptomic, proteomic, or metabolomic findings).
Define Study Type	Specify whether the study is prospective or retrospective.
Precisely Define Phenotype of Participants	Describe the clinical characteristics of T2DM patients, including disease duration, medication use, and glycemic control.
Carefully Select and Describe Controls	Include healthy controls and/or individuals with prediabetes. Ensure they are matched for age, BMI, and other relevant factors.
Calculate Sample Size and Power	Perform power analysis to determine adequate sample size for detecting significant differences or associations.
Provide Adequate Participant Data	Document age, sex, BMI, ethnicity, duration of diabetes, HbA1c levels, comorbidities, and medication history.
Assess Environmental and Lifestyle Factors	Record data on diet, physical activity, smoking, alcohol consumption, and socioeconomic status.
Identify Risk Factors and Possible Confounders	Identify Risk Factors and Possible Confounders
Design Patient Informed Consent	Design Patient Informed Consent

**Table 2:** Considerations for sample collection and preservation across different 'Omics' platforms in type 2 diabetes mellitus research.

‘Omics’ Platform	Sample Types	Considerations
Genomics (DNA)	Blood, Saliva, Pancreatic tissue	Stable at room temperature but best if refrigerated or frozen.
Epigenomics (DNA, RNA)	Blood, Pancreatic tissue	Require proper collection, meticulous processing, and sample storage at −80°C to ensure sample integrity.
Transcriptomics (RNA)	Blood, Pancreatic islets, Adipose tissue	Require appropriate collection, careful processing, and storage at -80°C to ensure RNA integrity and quality for analysis.
Proteomics (Proteins)	Blood, Pancreatic islets, Adipose tissue, Urine	Require rapid sample preparation and preservation (± protease inhibitors) prior to storage at −80°C to prevent non-specific protein degradation.
Metabolomics (Metabolites)	Blood, Urine, Adipose tissue	Require rapid metabolic ‘quenching’ (flash freezing or acid precipitation) to prevent degradation of metabolites.

## 4 Natural characterization of biomarkers in glycolipid metabolism research of type 2 diabetes mellitus

### 4.1 Genomics

In the exploration of the clinical research efficacy mechanisms of T2DM, it is imperative to commence with its genetic background, wherein genomics plays a crucial role. GWAS have played a pivotal role in unveiling the genetic diversity of T2DM, particularly through the DIAMANTE Consortium’s multi-ancestry GWAS, which involved over 1.3 million participants and identified 237 significant loci, thereby enhancing the translation of genetic discoveries across diverse populations (23). Furthermore, through the application of recessive genetic models, uncovering low-frequency but significantly impactful genetic variations. This has offered novel perspectives for understanding the complex genetic background and pathophysiological mechanisms of T2DM (24). The identification of these genetic loci has paved the way for the development of more precise tools for risk assessment, such as combining polygenic risk scores with tools like QDiabetes. Researchers can now not only identify different clinical subgroups at diagnosis but also more accurately predict the risk of T2DM across populations (25), underscoring the importance of genetic loci in disease progression and the necessity of multi-ancestry studies to better understand and address the global challenges of T2DM.

Considering that many genetic signals of T2DM manifest through dysfunction in pancreatic islet cells, a series of genomic explorations were conducted on human islets to identify candidate genes linked to the pathophysiology of T2DM. CRISPR-Cas9 technology facilitates precise genome editing by guiding RNA sequences to specific DNA targets, making it invaluable for modeling T2DM-associated mutations. This approach enables researchers to validate the functional impacts of genetic variants implicated in T2DM pathogenesis, providing insights into disease mechanisms and potential therapeutic targets (26). A study using whole-genome CRISPR screening revealed the potential role of autophagy receptors like CALCOCO2 in β-cell dysfunction, further enhancing our understanding of insulin content regulation mechanisms (27). Additionally, recent genomic research on Chinese patients with T2DM has highlighted the potential of leukocyte telomere length as a biomarker for blood sugar progression, revealing factors beyond the single-cell level impacting T2DM development (28). Importantly, recent advancements in genetic clustering and the development of process-specific polygenic scores have significantly enhanced the understanding of T2DM pathophysiology. As an example, Bayesian non-negative matrix factorization have enabled the stratification of T2DM-associated genetic loci into distinct clusters, each linked to specific pathophysiological mechanisms and clinical outcomes (29). This approach not only refines genetic insights but also aids in the development of targeted therapeutic interventions, propelling forward the precision medicine approach in managing T2DM (29). In terms of adipose tissue, a GWAS in the GENiAL cohort revealed a complex relationship between adipocyte numbers and the risk of T2DM (30). This study identified genetic loci associated with adipocyte numbers, finding overlaps with known T2DM risk loci. Moreover, multi-ancestry exome sequencing studies have provided new insights into the relationship between fat distribution and T2DM prevention. Particularly, large-scale sample analyses from the UK, Sweden, and Mexico identified 16 genes significantly associated with fat distribution (31). These discoveries not only emphasize the importance of body fat distribution as a major genetic risk factor for cardiometabolic diseases but also reveal that the impact of rare coding variants in fat distribution on disease risk is greater than that of common alleles, which is crucial for understanding the genetic mechanisms of the disease and developing targeted therapeutic strategies (31).

Recent studies employing genomic methods have provided profound insights into the genetic and phenotypic correlations between T2DM and its complications. A study analyzing 4,375 patients with T2DM observed a significant gradient relationship between the levels of mid-regional pro-adrenomedullin (MR-proADM) in plasma and the risk of lower limb amputation in diabetic patients, underscoring the potential of MR-proADM as a prognostic biomarker (32). Additionally, allelic variations in the ADM gene are associated with elevated levels of MR-proADM and an increased risk of lower limb amputation. Furthermore, some studies have shifted focus to the development trajectory and gene expression profiles of other diseases related to diabetes. For instance, in-depth analysis of sequential liver biopsy samples from patients with NAFLD revealed a significant correlation between elevated levels of HbA1c and the progression of liver fibrosis, independent of BMI (13). Gene set enrichment analysis further identified gene pathways associated with liver fibrosis progression and increased HbA1c levels, involving hepatocytes in zone 3 of the liver, liver sinusoidal endothelial cells, stellate cells, and plasma cells, thus offering new targets for future therapeutic strategies. Similarly, another large-scale study based on genomic data from the UK Biobank observed a positive genetic correlation between T2DM and stroke (14).

### 4.2 Methylation

Recent studies have delved into the patterns of DNA methylation, highlighting the central role of this epigenetic mechanism in the pathogenesis of T2DM and its detailed impact at the microscopic level of disease mechanisms. In examining DNA methylation patterns in T2DM, our initial focus was on the disease’s novel classification. A study investigated whether there were differences in DNA methylation among newly classified T2DM subtypes, including severe insulin-deficient diabetes, severe insulin-resistant diabetes, mild obesity-related diabetes, and mild age-related diabetes (33). By conducting whole-genome DNA methylation analysis on blood samples from newly diagnosed T2DM patients, researchers established Methylation Risk Scores (MRS) unique to each subtype. These MRS were significantly associated with different subtypes and the risk of future complications. Notably, the MRS for mild obesity-related diabetes was linked to a reduced risk of cardiovascular and renal diseases, whereas MRS for severe insulin-resistant diabetes and mild age-related diabetes were associated with an increased risk of these complications. This not only provides a basis for understanding the different subtypes of T2DM but also reveals the variability in DNA methylation among these subtypes, supporting the reclassification of diabetes and the need for precision medicine in T2DM subtypes (33).

Subsequently, our focus shifted to the DNA methylation studies in adipocytes, revealing the pivotal role of adipose tissue in the development of T2DM. One study analyzed DNA methylation patterns in blood samples from healthy individuals and patients with obesity and T2DM, identifying specific methylation markers significantly associated with disease states (34). Specifically, these studies pointed out increased or decreased methylation levels in certain gene regions in obese and T2DM patients, potentially affecting the expression of relevant genes and thus participating in the pathogenesis of obesity and T2DM. Moreover, another study utilizing subcutaneous adipose tissue samples from participants in the TwinsUK cohort, employed high-throughput techniques to reveal the complexity of DNA methylation patterns in adipose tissue, particularly their association with fat metabolism and insulin resistance. This study was validated and replicated in three independent cohort samples (35). Employing high-throughput technologies, such as the 450k Illumina HumanMethylation450 BeadChip and RNA-seq data, the study uncovered certain methylation sites associated with altered adipocyte function and metabolic pathways, suggesting these methylation changes could be key factors in the pathogenesis of obesity and T2DM. This Next-Generation Sequencing technology allows for the comprehensive analysis of genomic variations implicated in T2DM, including rare variants that contribute to disease susceptibility and progression (36). From the novel classification of T2DM to the DNA methylation studies in adipocytes, we not only uncovered the methylation differences between different subtypes but also identified specific methylation markers significantly associated with disease states.

### 4.3 Transcriptomics

Transcriptomics plays a pivotal role in revealing the gene regulatory abnormalities and cellular dysfunctions in complex metabolic disorders such as T2DM. In the quest to elucidate the pathophysiological underpinnings of T2DM, recent transcriptomic analyses have significantly advanced our understanding of β-cell dysfunction, a central element in the disease’s progression. A pivotal study investigated the gene expression profiles in pancreatic islets from around 300 individuals, identifying 395 differentially expressed genes associated with T2DM (37). Notably, this study uncovered both novel and previously recognized candidate genes, including PAX5 and OPRD1, which are linked to impaired insulin secretion and mitochondrial dysfunction in β cells (37). In addition, single-cell RNA sequencing has revealed the heterogeneity of β-cell subpopulations in T2DM, such as subgroups with high CD63 expression associated with increased mitochondrial metabolic gene expression, suggesting their important roles at different stages of the disease (38). Strategies to reconstitute or maintain CD63hi beta cells may represent a potential anti-diabetic therapy. Single-cell analysis techniques have demonstrated their powerful capabilities in T2DM β-cell studies, revealing the complexity and dynamic changes in the significant heterogeneity within β-cells. For instance, a study of 34 individuals across non-diabetic, pre-diabetic, and T2DM stages, combining single-cell transcriptomics (scRNA-seq) with chromatin accessibility measurements, elucidated specific mechanisms of β-cell dysfunction and the changes in β-cell subpopulations during disease progression (39). Moreover, an innovative study utilizing single-cell and single-nucleus RNA sequencing (snRNA-seq) delved deeply into the subpopulations of human pancreatic β-cells, revealing their dynamic biological behaviors in various environments (40). This study found significant changes in gene expression patterns of β-cells *in vitro* and in transplant environments, suggesting potential pathways for functional changes in β-cells in T2DM (40). Additionally, another study integrating single-cell transcriptomic and epigenomic data revealed the transcriptional heterogeneity in T2DM pancreatic β-cells, focusing particularly on the downregulation of HNF1A gene expression and its impact on β-cell function (41). This study underscored the importance of the HNF1A gene in maintaining β-cell electrical activity and functionality, finding its downregulation closely linked to T2DM progression. These findings collectively point to a core concept: the heterogeneity of T2DM pancreatic β-cells is not only manifested in gene expression but also reflected in their response to internal and external environmental changes. This in-depth understanding from the molecular to the cellular level provides a new perspective on the pathophysiological mechanisms of T2DM. Concurrently, transcriptomic analysis has revealed functional changes and heterogeneity in α-cells under diseased states, potentially exacerbating glucose control issues. A study combining electrophysiological recording with single-cell RNA sequencing revealed transcriptional differences in α-cells with high and low ARX expression (42). Additionally, another study employing 3D chromatin architecture to dissect cell-type-specific gene regulatory programs delved into the regulatory structures of T2DM risk at the cellular level (43). By generating transcriptomic and 3D epigenomic landscapes of human pancreatic acini, α, and β cells, the study highlighted the cell-type stratified connections between candidate causal variants and their target effector genes (43). These studies emphasize the adaptation and dysfunction of β-cells and α-cells in the pathological changes of T2DM, becoming one of the core mechanisms of the disease’s onset. Further exploration of the molecular mechanisms in human pancreatic islets underscores the complexity of islet cells in T2DM. The role of ERRγ in pancreatic acinar cells highlights a broader functional regulatory network of islet cells. A key study demonstrated that functional inhibition of ERRγ in mouse models led to a pancreatitis-like phenotype and cell death, while transcriptomic alterations associated with reduced ERRγ expression were observed in clinical samples from patients with chronic pancreatitis (44). Additionally, a recent study analyzed 399 human islet samples using transcriptomic methods such as principal component analysis, gene expression quantification, splicing quantification, and cis-eQTL and cis-sQTL mapping, revealing the critical role of islet RNA splicing in genetic regulation (45). In the study of T2DM and its associated systemic insulin resistance, a multi-omics study involving 70 obese patients underscored the key role of transcriptomics in revealing insulin resistance mechanisms in adipose tissue (46). This study conducted an in-depth transcriptomic analysis of abdominal subcutaneous and visceral fat samples, comparing gene expression differences between insulin-sensitive and insulin-resistant patients (46). The results revealed specific transcriptional changes in adipose tissue under insulin-resistant conditions, reflecting functional impairments in adipocytes in insulin signaling, as well as disturbances in molecular pathways during energy metabolism and inflammatory processes. Furthermore, integrating methylation and proteomics analyses, the study further elucidated the regulatory mechanisms behind these transcriptional changes, providing a more comprehensive perspective on the molecular mechanisms of insulin resistance. T2DM is a systemic metabolic disorder, characterized not only by impaired glucose-regulating functions of islet cells but also by metabolic pathway alterations in other tissues, such as the skin (47). For instance, studies on autophagic processes in skin samples from T2DM patients emphasized the critical role of N6-methyladenosine RNA modification in cellular stress responses, particularly through the effects of YTHDC1 protein on SQSTM1/p62 mRNA. This cross-tissue molecular perspective offers a more comprehensive biological picture of T2DM, thereby aiding in revealing the holistic nature and therapeutic potential of this complex disease (47).

### 4.4 Proteomics

Proteomics plays a crucial role in elucidating the biological processes of T2DM and enhancing the accuracy of disease prediction. Mass Spectrometry (MS), including liquid chromatography-tandem mass spectrometry (LC-MS/MS), plays a crucial role in proteomic studies by accurately identifying and quantifying proteins, enhancing biomarker discovery in T2DM through sensitive detection of disease-related protein signatures. For instance, A study utilizing LC-MS/MS explored the interaction network of TBC1D4 protein in human skeletal muscle, particularly under insulin stimulation, revealing key regulatory pathways of insulin sensitivity and glucose uptake in muscle (48). These studies demonstrate the application of proteomics in elucidating insulin signaling pathways and highlight its significance in uncovering the mechanisms of T2DM, providing deeper insights into the complex network of insulin signaling for advancing T2DM research and treatment. Moreover, a long-term follow-up study, spanning 18 years, analyzed data from 2,631 participants in the Cardiovascular Health Study and 752 participants in the HERITAGE Family Study, identifying 51 proteins associated with T2DM risk (49). These proteins, including newly identified ones like β-glucuronidase and plexin-B2, are linked not only to diabetes risk but also to key physiological indices such as insulin sensitivity index, acute insulin response, and glucose effectiveness (49). Another study on a targeted proteomics approach in a South African Black population further deepened our understanding of insulin sensitivity and β-cell function (50). Analyzing plasma samples from 380 men and 375 women for a predefined set of 184 proteins, the study revealed proteins related to insulin dynamics, such as IGFBP2 and TIMP4, emphasizing the gender specificity in diabetes pathogenesis (50).

Subsequently, the KORA study provided a pathophysiological view from cellular to systemic levels by analyzing proteomic changes from prediabetes to T2DM (51). By examining 233 proteins in plasma samples from 1,653 participants, the study identified proteins related to prediabetes, newly diagnosed T2DM, and T2DM prevalence (51). These findings include proteins like IL-17D and IL-18 receptor 1, some of which are considered new candidates for treatment. Additionally, a study on African Americans utilized high-throughput proteomics to identify 325 proteins closely associated with T2DM progression (52). These proteins reflect the complex biological processes of diabetes, going beyond traditional markers like obesity, blood glucose, and insulin resistance (52). Notably, even after accounting for factors like BMI and fasting blood glucose, 36 of these proteins still showed significant correlations, further revealing their potential role in diabetes development. Another study identified 47 plasma proteins predicting the onset of T2DM, some with causal roles related to inflammation and lipid metabolism pathways (53). Meanwhile, the application of proteomics in multi-omics predictive studies has also demonstrated its indispensable significance. A comprehensive study, integrating genomics, proteomics, and metabolomics, predicted the risk of T2DM onset (54). The results indicated that protein biomarkers showed the highest predictive performance compared to genomic or metabolomic levels alone (54). Recent Mendelian randomization studies further revealed circulating proteins causally linked to adult and adolescent-onset T2DM, offering new insights for early identification and treatment (55). Notably, proteins such as C-type mannose receptor 2, MANS domain-containing protein 4, and sodium/potassium-transporting ATPase subunit beta-2, play significant roles in the pathological process and provide new directions for potential drug target research in T2DM (55).

In exploring T2DM complications, a study focused on the tumorigenic neuroblastoma suppressor protein 1 finding its concentration significantly associated with glomerular damage in T2DM nephropathy, offering a new molecular perspective for understanding T2DM renal complications (15). Another study performed Mendelian randomization and colocalization analysis on large-scale population plasma proteomes, identifying key proteins associated with T2DM and its complication risks (56). These proteins, including HLA-DRA, AGER, HSPA1A, and HSPA1B, are related to various microvascular complications, revealing pathogenic proteins in T2DM and its complications, thus enhancing our understanding of their molecular etiology.

### 4.5 Metabolomics

Metabolomics is particularly important in exploring the complex biological background of T2DM as it can precisely capture early signs of metabolic dysregulation, often present before the manifestation of clinical symptoms. Specifically, analyzing the metabolic profiles in clinical samples allows researchers to deeply understand the pathophysiology of T2DM, including but not limited to lipids, carbohydrates, amino acids, and nucleic acids. These subtle changes in metabolites not only reveal the molecular mechanisms of the disease but also provide new perspectives for diagnosis, treatment, and prevention.

Chromatography–MS platforms are frequently used to provide the sensitive and reproducible detection of hundreds to thousands of metabolites in a single biofluid or tissue sample, including applying gas chromatography-MS (GC-MS) and ultraperformance liquid chromatography-MS (57). In the field of lipidomics, changes in specific phospholipid molecules or fatty acid compositions have been found to potentially indicate early disease onset or serve as indicators of therapeutic efficacy. For example, to compare with the concentrations of polyunsaturated fatty acids derived from the lipidomics analyses by MS, the EPIC-Potsdam prospective cohort measured polyunsaturated fatty acids in total plasma phospholipids by GC-MS with a flame ionization detector. The study revealed a potential link between specific n-6 polyunsaturated fatty acid concentrations in plasma and T2DM risk (58). Further, the application of metabolomics is not limited to understanding disease mechanisms; it also plays a significant role in disease risk assessment. A study utilizing lipidomics data from two large Australian population cohorts conducted an in-depth analysis of the metabolic phenotypes of BMI. The study revealed significant differences in T2DM-related metabolic dysregulation among individuals with similar BMI (59). This finding highlights the potential of lipidomics analysis in identifying individuals at risk for T2DM, despite having a normal BMI. Additionally, a recent study combined shotgun lipidomics with a machine-learning-based model, reporting the application of lipidomics in predicting the incidence of diabetes and cardiovascular diseases in populations. This study is evidently notable in demonstrating the role of lipidomics in disease prediction and translational research (60). Furthermore, the application of metabolomics extends beyond lipid analysis. Through prospective analysis of a wide array of metabolites, research has revealed various metabolites associated with T2DM risk, such as specific amino acids, carbohydrates, acylcarnitines, and glycerides (61). Notably, researchers identified biomarkers of disease progression in three T2DM cohorts, covering metabolites, lipids, and proteins across 2,973 individuals (62). Integrative analysis led to the discovery of several key metabolites associated with worsening glycemia. Notably, Homocitrulline, isoleucine, and 2-aminoadipic acid, eight triacylglycerol species, and lowered sphingomyelin 42:2;2 levels were identified as markers of faster disease progression (62).

In the application of metabolomics, challenges in clinical relevance and data interpretation are observed. Despite the identification of numerous potential biomarkers, their clinical significance and application remain complex issues. These challenges include the reproducibility, robustness, and universality of biomarkers across different populations. Hence, subtype analysis for diverse populations becomes particularly important. For instance, a comprehensive study using Qatar Biobank data, encompassing sociodemographic, clinical, and behavioral phenotypes, provided new perspectives. In this study, researchers used the aptamer-based SOMAscan platform and Metabolon HD4 technology to measure levels of 1,305 circulating proteins in 2,935 samples and 1,159 metabolites in 3,000 samples (17). In non-targeted metabolomics applications, studies in various populations have identified specific metabolites related to T2DM incidence. In a study among Hispanic Americans, analyzing 624 known serum metabolites, 134 were found to be associated with T2DM incidence, revealing unique T2DM onset mechanisms related to diet and hormone levels, such as androgenic steroid modules increasing diabetes risk (18). Similarly, a study in African Americans, detecting over 2,600 compounds, contributed to a comprehensive understanding of T2DM metabolites in different ethnic groups and improved diabetes prediction models (63). Additionally, a study in the Swedish population, using non-targeted ultra-high-performance liquid chromatography and time-of-flight MS, discovered specific serum metabolites, like hexanoylcarnitine and tryptophan, not only associated with T2DM incidence risk but also significantly improving existing disease mortality prediction models (64).

In exploring the role of metabolomics in understanding the pathogenesis of T2DM complications, key studies have provided deep insights. Firstly, a study on renal injury in an endogenous purine-mediated diabetes model utilized multiple clinical cohorts and comprehensive experimental methods, including urinary metabolomics, MALDI-MS imaging of human kidney biopsies, animal model studies, and cell cultures, to investigate the role of purines in diabetic nephropathy (65). The study found that the adenine/creatinine ratio, a urinary biomarker, is closely related to the development of end-stage kidney disease. Moreover, based on two large randomized clinical trials, ACCORD and VADT, researchers explored the association between advanced glycation end-products and renal function loss through LC-MS/MS analysis of blood and urine samples (66). The study discovered that specific advanced glycation end-products in serum and plasma are significantly associated with declining renal function and chronic kidney disease risk in T2DM patients. On the other hand, the Joslin Kidney Study team conducted global serum metabolomics analysis using the Metabolon platform in 409 T2DM patients. Through logistic regression analysis, researchers evaluated the association between Cardiovascular Disease events and 671 metabolites and performed absolute quantification of significant metabolites in a validation set from the Joslin Heart Study (67). This study revealed specific metabolites associated with cardiovascular disease risk in T2DM patients. These studies demonstrate that metabolomics technology can provide a comprehensive view of lipid metabolism disorders in T2DM and help identify new biomarkers and therapeutic targets.

### 4.6 Microbiome

In exploring the relationship between the development of T2DM and the gut microbiome, a series of studies have emphasized the intricate interplay between intestinal microbes and host metabolism. The 16S rRNA gene has been a mainstay of sequence-based bacterial analysis for decades (68). Recent studies using 16S rRNA gene sequencing analysis of human fecal samples identified a novel symbiotic bacterium, Dysosmobacter welbionis, commonly present in healthy individuals and negatively correlated with body mass index, fasting glucose, and glycosylated hemoglobin in obese individuals with metabolic syndrome (69). Further researchers analyzed fecal metabolites from 142 individuals with impaired fasting glucose and 1,105 healthy individuals from the TwinsUK cohort, as well as a replication study using the KORA cohort, identifying 8 metabolites positively correlated with impaired fasting glucose, including 1-methylxanthine and niacin (70). The studies also discovered that specific intestinal microbes, such as Faecalibacillus intestinalis and Dorea formicigenerans, were strongly correlated with these host metabolites, suggesting a potential role of gut microbiota in modulating the intestinal absorption/excretion of host metabolites and xenobiotics, thus relating to the risk of prediabetes (70).

In the field of T2DM research, metagenomic sequencing has emerged as a key tool for exploring the relationship between gut microbiota and disease progression. This technique allows for a comprehensive analysis of the microbial species present in the gut and their functions, revealing complex interactions between these microbes and host metabolic processes. For instance, a study found that specific butyrate-producing bacterial groups, like Coprococcus, were associated with higher insulin sensitivity and reduced T2DM risk, while other groups, such as Flavonifractor, were linked to decreased insulin sensitivity and increased T2DM risk (71). Furthermore, the analysis of antibiotic resistance genes has shed light on the connection between microbiome composition and disease risk. A study focused on human intestinal antibiotic resistance genes used metagenomic sequencing to analyze the gut microbiota of healthy individuals, those with prediabetes, and T2DM patients (72). This research found significant differences in the gut antibiotic resistance gene composition between T2DM patients and both healthy and prediabetes groups, suggesting a link between antibiotic resistance gene diversity and increased T2DM risk (72).

In a study that conducted an in-depth analysis of clinical samples from patients with T2DM and Metabolism-Associated Fatty Liver Disease (MAFLD) using microbiomics, clinical data from 20 MAFLD patients, 20 patients with both MAFLD and T2DM, and 19 healthy donors were analyzed (73). Microbiome and metabolomic analyses revealed significant similarities and differences in clinical indicators (such as BMI, ALT, PCHE, CAP) and liver metrics between the two patient groups compared to the healthy control group. Microbiome and bioinformatics analysis showed significant differences in gut microbial characteristics (such as abundance, phylogenetic analysis, species) and lipid metabolism (such as metabolites, correlation coefficients, and scatter plots) across the groups. Notably, certain highly expressed microbes (such as Elusimicrobiota, Berkelbacteria, Cyanobacteria, Peregrinibacteria) and lipid metabolites (such as Lipid-Q-P-0765) were identified. These findings may aid in the clinical diagnosis of MAFLD and T2DM.

### 4.7 Molecular biology techniques

The application of various biotechnological methods has significantly deepened our understanding of the pathophysiology of T2DM. Studies focusing on the molecular mechanisms behind β-cell dysfunction have explored the causes and manners of β-cell damage in T2DM. For instance, research has revealed key changes in intracellular signaling pathways within β-cells, such as the downregulation of Phosphatidylinositol Transfer Protein Alpha during the maturation process of insulin granules, underscoring the importance of specific molecular pathways in β-cell dysfunction (74). Additionally, a study emphasized the role of palmitoylation in human islet cells. Employing antibody techniques to detect the expression of DHHC6 in islet cells and conducting insulin secretion experiments, the study associated DHHC6 palmitoylation with β-cell hyperactivity and exhaustion (75). Moreover, an innovative approach inducing adult pancreatic β-cell regeneration by disrupting the DREAM complex highlighted a novel therapeutic strategy aimed not just at controlling blood glucose levels but at restoring β-cell function (76). Utilizing a range of techniques, such as viral transduction, islet dispersion, proximity ligation assays, and qPCR, this study provided valuable insights into mechanisms supporting β-cell replication and insulin production recovery (76).

Recent studies collectively demonstrate the multidimensional mechanisms of metabolic dysregulation in prediabetes and T2DM. Specifically, a study involving 86 normal-weight or obese volunteers, and 25 obese volunteers before and after weight loss, explored the relationship between adipose tissue inflammation and insulin resistance by measuring insulin concentration (77). This study found that the size of femoral fat cells is a strong predictor of adipose tissue insulin resistance and lipolysis, while common AT inflammation markers not significantly associated with AT insulin resistance offered new interpretations of the relationship between prediabetes and insulin resistance. Additionally, an innovative study using microfluidic co-culture chips provided insights into the complex interactions between the pancreas and liver in the metabolic response of prediabetes. The study, employing 3D-cultured human liver organoids and islet cells and integrating techniques like Liquid Chromatography-Tandem MS, quantitative PCR (qPCR), and Western blotting, comprehensively analyzed metabolic activity, gene expression profiles, and insulin sensitivity. These findings offer new perspectives on early metabolic changes in prediabetes, emphasizing the utility of bioengineering systems in elucidating human metabolic disorders (78).

In exploring biomarkers of T2DM, recent studies have jointly revealed the complexity of metabolic abnormalities at the molecular level in T2DM and their systemic impacts. For example, an intriguing study explored the autoimmune response to PDIA3 epitopes and its association with liver damage in T2DM patients (79). Through analyses of human samples and mouse models, the study illuminated the role of PDIA3 in autoimmune hepatitis and primary biliary cholangitis in diabetic patients. This research not only deepened our understanding of liver complications in T2DM patients but also highlighted the complex impact of the disease on the immune system and metabolic health. Another study expanded this concept, shifting focus from metabolic abnormalities to the application of stem cell therapy in improving microangiopathy in T2DM patients (80). Researchers extracted stem cells from the human body, inducing their differentiation into specific mesodermal cell subsets, and then studied their effects on microangiopathic changes in Type 2 diabetic animal models. These findings not only demonstrated the cells’ ability to ameliorate the microangiopathic state in animal models but also revealed the profound impact of the disease on the microcirculatory system.

In genetics and epidemiology, a study on the South Asian Indian population provided epidemiological and genetic perspectives, using a cross-sectional design to compare the prevalence of T2DM in healthy, overweight, and obese participants. This study found that the prevalence of young-onset diabetes in Asian Indians was significantly higher than in European Caucasians, even at normal BMI levels. The study also employed partitioned polygenic scoring to assess the genetic determinants of β-cell function, finding that the genetically determined β-cell function in Asian Indians was lower than in European Caucasians (81). This study highlights critical genetic and racial susceptibilities, prompting consideration of more targeted screening and preventive measures for specific populations.

## 5 Omics technologies in the study of glycolipid metabolic interventions in type 2 diabetes mellitus

### 5.1 The interplay of genomics, epigenomics, and transcriptomics in T2DM glycolipid metabolism research

Recent studies have highlighted the critical role of gene expression and molecular regulation in exploring the glycolipid metabolic mechanisms and pharmacological interventions of T2DM. Initially, a study within the Diabetes Prevention Program revealed genetic variations influencing the response to metformin (82). This study, through GWAS, identified specific variations near ENOSF1 and OMSR, closely associated with levels of glycated hemoglobin and body weight changes, emphasizing the importance of genetic factors in modulating drug responses and metabolism (82). Building on this, another study delved into the molecular regulation of Pioglitazone (a PPARγ agonist), examining its impact on adipose tissue and the circulating extracellular vesicles (EV) containing microRNAs. By measuring levels of EV-miRNAs from adipocytes in plasma, this study further deepened our understanding of intercellular communication and metabolic regulation mechanisms in T2DM (83). Results showed that, compared to placebo, Pioglitazone treatment significantly downregulated 5 types of miRNAs in EVs (including miR-7-5p, miR-20a-5p, miR-92a3p, miR-195-5p, and miR-374b-5p), while the expression trend of miR-195-5p was opposite in subcutaneous adipose tissue. These changes in miRNA expression were associated with inhibition of lipolysis and improved insulin sensitivity (83).

In exploring the complex network of T2DM and its impact on glycolipid metabolism, epigenomic technologies have shed light on the multifaceted effects of dietary interventions, from adults to children, from individual responses to intergenerational transmission. In the POUNDS Lost trial, the study focused on individual differences in DNA methylation in the ABCG1 region and their response to dietary weight-loss intervention, particularly in terms of long-term fat mass and fat distribution changes (84). Using high-resolution methylation C capture sequencing, the results identified the ABCG1 gene as closely related to lipid metabolism, especially in relation to changes in protein intake (84). These findings emphasize the necessity of considering genomic characteristics in formulating dietary plans for T2DM patients, revealing the potential of genome-marker-based personalized nutrition strategies in improving T2DM treatment outcomes. Additionally, another study investigated the metabolic consequences of late-night calorie intake in overweight and obese adults, using microarray technology and RNA-Seq to analyze gene expression in subcutaneous abdominal adipose tissue and blood samples (85). Indirect calorimetry and polysomnography confirmed these findings, revealing how meal timing significantly affects metabolic gene expression and energy balance. Building on this understanding in adults, scientists further explored how the dietary glycemic index and glycemic load affect metabolic health in children and adolescents through DNA methylation, particularly the differences in these relationships in overweight and obese individuals (86). Through an Environmental Genome-Wide Association Study of 1,187 children and adolescents, the study found dietary GL significantly correlated with methylation at specific CpG sites, closely associated with gene expression related to metabolic dysfunction. These findings not only reveal the potential epigenetic impact of high glycemic index and glycemic load diets on metabolic health in young populations but also highlight the specificity of this impact in the context of weight gain. Further, researchers expanded their exploration from the current generation to intergenerational transmission, studying the impact of maternal diet during pregnancy on offspring umbilical cord blood DNA methylation, providing new dimensions to understand the intergenerational transmission of T2DM risk factors (87). Through Environmental Genome-Wide Association Study of umbilical cord blood DNA from 2,003 mother-offspring pairs across three cohorts, the study revealed the impact of maternal glycemic characteristics and BMI on offspring DNA methylation patterns and how these impacts relate to birth weight and child BMI. This study not only highlights the far-reaching influence of maternal health status on offspring metabolic health but also offers deeper insights into understanding the complexity and multilayered nature of T2DM and its associated glycolipid metabolic issues.

In exploring therapeutic strategies for T2DM complications, recent genomic and transcriptomic studies have provided new insights. A study focusing on diabetic nephropathy employed RNA sequencing and immunostaining techniques to deeply analyze the association between tubular p21 gene expression, DNA damage, and cellular aging. Elevated expression of p21 was observed in both diabetic mouse models and human kidney tissue samples, suggesting a potential role in the progression of diabetic nephropathy (88). Further pharmacological intervention studies, such as the use of SGLT-2 inhibitors, confirmed the potential of reversing diabetic nephropathy by regulating p21 expression (88). Another study focused on the role of the APLN/APJ signaling pathway in T2DM complications, particularly in regulating testicular function (89). Through transcriptomic analysis, this research revealed the crucial role of the APLN/APJ pathway in T2DM-related pathological processes, providing clues for new therapeutic targets. In a study on reproductive dysfunction in patients with T2DM, the APLN receptor antagonist ML221 was used as a therapeutic intervention to experimentally treat testicular pathological changes in T2D patients (89). The study first conducted in-depth analysis of testicular samples from diabetic patients using single-cell transcriptomic sequencing technology, revealing significant damage to Sertoli cell function and the blood-testis barrier. Subsequent application of ML221 in diabetic mouse models successfully reversed these pathological changes, significantly improving BTB structural damage and spermatogenesis (89). These experimental results not only confirmed the key role of the APLN/APJ axis in reproductive dysfunction in T2D patients but also demonstrated the potential application of pharmacological interventions targeting this axis in improving reproductive function.

### 5.2 Proteomics

In exploring the treatment and management of T2DM, the contribution of proteomics is increasingly prominent, especially in understanding molecular dynamics and their response to intervention measures. For instance, a study in the SLIMM-T2D clinical trial utilized proteomic sequencing technology and pathway enrichment analysis to explore the molecular impact of weight-loss surgery on T2DM control (90). The study revealed the key role of the Growth Hormone Receptor pathway in metabolic improvement after Roux-en-Y gastric bypass. This was validated in human samples and further confirmed through rodent models. The study emphasized the reduction in Growth Hormone Receptor and its impact on changes in insulin-like growth factor-binding proteins and plasma Growth Hormone levels, providing in-depth insights into the biological mechanisms leading to diabetes remission post-surgery. Another study from the DiRECT and DIADEM-I randomized clinical trials used the SomaSignal predictive test, based on extensive measurements of plasma proteins, to study the impact of significant weight loss on cardiac metabolic health (91). The research found that weight loss was significantly associated with improvements in cardiac metabolic health indicators, such as liver fat, glucose tolerance, and cardiopulmonary health, especially when the weight loss exceeded 10 kilograms, significantly reducing cardiovascular risk.

These studies collectively highlight the core role of proteomics in deeply deciphering and accurately predicting the effects of T2DM treatment interventions, covering a range of interventions from dietary adjustments to weight-loss surgery. They underscore the capability of proteomic analysis in identifying key molecular mechanisms in T2DM treatment and assessing intervention effects, significantly advancing our goal of achieving personalized medicine in the comprehensive management of T2DM.

### 5.3 Metabolomics

In the study of glycolipid metabolism in T2DM, the integration of metabolomics technology with various strategies such as pharmacological interventions, weight management, and dietary interventions has not only revealed the complexity of metabolic changes but also deepened our understanding and influence on disease progression. For instance, a study utilized sodium phenylbutyrate to modulate branched-chain amino acid metabolism in T2DM patients, analyzing metabolic changes in 18 T2DM patients after sodium phenylbutyrate treatment through a randomized, double-blind, placebo-controlled crossover design (92). The findings highlighted significant improvements in metabolic health, especially in enhancing peripheral insulin sensitivity and reducing plasma levels of BCAAs and glucose. Extending to the realm of weight management, the DiRECT study employed high-throughput metabolomics technology to focus on the impact of weight loss and diabetes remission on the metabolome (93). Analyzing serum samples from 298 T2DM patients, the study found significant metabolomic changes induced by the weight management program compared to conventional guideline treatments. These changes included reduced levels of branched-chain amino acids, sugars, and triglycerides in low-density lipoprotein, as well as increased levels of sphingolipids, sphingomyelins, and fatty acid metabolism-related metabolites. Particularly in individuals achieving significant weight loss and diabetes remission, the reductions in glucose, fructose, and mannose levels were more pronounced, underscoring the close association between metabolomic changes and clinical efficacy.

Within this framework, a significant trend is the use of metabolomics technology to deeply understand how different dietary patterns affect glycolipid metabolism and disease risk. Plant-based diets, particularly rich in dietary fiber, have shown special importance in regulating metabolic health. For example, a study analyzing plasma metabolites of participants in the Nurses’ Health Study and Health Professionals Follow-Up Study found a significant association between plant-based diets and reduced T2DM risk (94). Utilizing high-throughput LC-MS technology, the study revealed a metabolomic profile associated with adherence to a plant-based diet and found these metabolites inversely related to T2DM events. Notably, metabolites like trigonelline, methylmalonate, and isoleucine played a significant role in mediating beneficial health effects, accounting for a large part of the diet-related reduction in diabetes risk (94). Furthermore, in a comprehensive study linking tryptophan metabolism to T2DM, researchers found that dietary fiber intake, rather than protein or tryptophan-rich foods, significantly influenced circulating tryptophan metabolites through complex interactions among host genetics, the gut microbiome, and diet. This interaction, particularly evident in lactase non-persistence individuals with higher milk intake, shifted tryptophan metabolism towards beneficial gut microbial indolepropionate production, highlighting potential personalized dietary strategies for T2DM management (95). In this context, the Mediterranean diet, another plant-based dietary pattern, similarly emphasizes the intake of fruits, vegetables, whole grains, and healthy fats like olive oil. In this study, researchers analyzed the lipidomic characteristics of the Mediterranean diet compared to traditional Chinese or transitional diets using high-throughput targeted LC-MS/MS, exploring how these dietary habits influence T2DM risk (96). These studies comprehensively showcase the importance of healthy dietary habits in optimizing metabolic health, revealing how good dietary patterns can effectively mitigate metabolic disorders and demonstrate how regulating key metabolites can reduce the risk of T2DM.

In exploring the impact of dietary patterns on the risk of T2DM, contrary to healthy diets, some studies focused on those dietary patterns that may increase T2DM risk. For instance, one study delved into the metabolic potential of inflammatory and hyperinsulinemic dietary patterns in T2DM risk. Using plasma metabolomics analysis and elastic net regression, this study identified specific metabolites associated with empirical dietary inflammatory patterns and indices linked to hyperinsulinemia. The results underscored a strong positive correlation between these dietary patterns and increased T2DM risk (97). Further, during the baseline recruitment period of the Hispanic Community Health Study/Study of Latinos, 2842 adult participants without diabetes, cardiovascular disease, or cancer were included (98). In this study, researchers used an untargeted approach to analyze fasting serum metabolomics, identifying eight metabolites inversely associated with various dietary scores. These metabolites showed harmful associations with insulin resistance—a key risk factor for T2DM (98). Through such research, we can gain a deeper understanding of how dietary choices affect insulin metabolism and its role in the onset of T2DM. This emphasizes the importance of adopting comprehensive dietary strategies in diabetes prevention and management to maintain metabolic health and control disease progression.

### 5.4 Microbiomics

In the exploration of therapeutic and management strategies for T2DM and its prediabetic states, the role of the gut microbiome has increasingly garnered attention. A series of studies have focused on the interaction between T2DM therapeutic drugs and the gut microbiome. Notably, in a study involving T2DM patients, researchers examined the effects of Acarbose and Vildagliptin, two antidiabetic medications, on the gut microbiome. The results showed that these drugs not only significantly improved glycemic control and visceral fat accumulation but also substantially altered the composition and metabolic pathways of the gut microbiota, closely linked to the therapeutic efficacy of the drugs (99). Additionally, the Food4Gut trial focused on the impact of the gut symbiont Dysosmobacter welbionis on metabolic health in obesity and T2DM, particularly examining the effects of Metformin and prebiotic treatments (100). In a study involving 106 participants, it was found that individuals treated with Metformin had a higher abundance of D. welbionis, which was associated with improved liver health and glucose metabolism (100). This finding suggests that Metformin may exert its antidiabetic effects by modulating specific gut microbes. Building on this, prebiotics not only provided a valuable contrasting perspective as a control group but also expanded this domain as a non-pharmacological treatment modality. For instance, a study in the MetaCardis cohort demonstrated that a strategy combining biotin and prebiotic supplementation could help prevent worsening metabolic status in severely obese patients (101).

As research into the impact of the gut microbiome deepens, some studies have begun to focus on the influence of lifestyle interventions(LSI) on gut microbial composition. Specific dietary changes have been identified as significantly related to species-level variations in the microbiome composition. Several microbes have been identified as mediators between specific dietary changes and clinical outcomes, including blood sugar control, high-density lipoprotein cholesterol, and triglycerides (102). For example, one study determined how a high-fat/high-sugar diet can damage white adipose tissue oxidation phosphorylation by altering gut microbiota and affecting macrophage activity, further linking inflammation to insulin resistance (103). This study also explored therapeutic interventions, showing that targeting MMP12 can improve glucose metabolism, highlighting new avenues for managing insulin resistance (103). Additionally, a key study examined the explicit link between sedentary behavior and adipocyte insulin resistance (104). In a comprehensive analysis involving 204 sedentary and 336 physically active participants, the effect of insulin on triglyceride hydrolysis and synthesis in subcutaneous adipocytes was assessed. Despite similar maximum hormonal effects in both groups, sedentary individuals showed a tenfold decreased sensitivity to insulin’s anti-lipolytic and lipogenic actions, indicating that a sedentary LSI is a significant factor in early pathophysiological changes leading to T2DM. While specific dietary changes, such as a high-fat/high-sugar diet, have been proven to exacerbate diabetes risk through changes in the gut microbiome, in contrast, healthy dietary interventions like personalized low-glycemic targeted diets and low-energy total meal replacements have shown the potential to improve metabolic health through positive regulation of the gut microbiome. These studies reveal that adjusting dietary intervention strategies can significantly impact the composition of the gut microbiome, thereby promoting metabolic health and alleviating the pathological states of prediabetes and T2DM. For example, in a personalized dietary intervention study in prediabetic patients, compared to a standard Mediterranean diet, a personalized postprandial glucose-targeted diet altered the host’s oral and gut microbiome, cardiac metabolic characteristics, and immune response, aiding in alleviating hyperglycemia and enhancing metabolic health (102, 105). Building on this, research has shifted to exploring the predictive ability of gut microbiome composition in response to dietary interventions, especially in terms of metabolic health and disease risk assessment. In a study involving 287 men analyzing stool and blood samples, the relationship between dietary habits and tryptophan metabolites was assessed (106). The study found that the abundance of 17 microbial species was significantly associated with plasma levels of indolepropionic acid, and a high tryptophan intake significantly increased indolepropionic acid levels against a high-fiber diet background (106). Furthermore, dietary and plasma tryptophan and its kynurenine pathway metabolites correlated positively with T2DM risk, while indolepropionic acid concentration was significantly associated with reduced T2DM risk. Similarly, in a study within the PREVIEW intervention framework, baseline gut microbiome composition was found to predict changes in body fat following a low-energy diet, further emphasizing the positive impact of a healthy diet on metabolic health in prediabetic patients (107).

In the field of T2DM treatment research, fecal microbiota transplantation (FMT) has garnered widespread attention as an innovative approach. For instance, a study involving the transplantation of gut microbiota from obese and T2DM female patients post-gastric or gastric bypass weight loss surgery into germ-free mice and specific pathogen-free rats found that post-surgery gut microbiota significantly improved glycemic control. This effect was associated with alterations in intestinal morphology and reduced absorption of glucose mediated by sodium-glucose cotransporter 1 (Sglt1), independent of changes in obesity, insulin levels, or insulin resistance (108). A groundbreaking study highlighted the role of gut microbiota in exacerbating intestinal permeability and inflammation, key factors in the progression of obesity and diabetes (109). Using fecal culture media and fecal microbiota transplantation, the study found that microbiota from obese mice and humans had diminished capacity to metabolize ethanolamine, leading to its accumulation in the intestines (109). This molecular alteration weakened the intestinal barrier, ultimately leading to increased gut permeability, inflammation, and glucose metabolism disorder (109). Notably, the study tested a new type of probiotic therapy aimed at restoring the ethanolamine metabolic capacity of the gut microbiota. This intervention effectively reduced increased intestinal permeability and related inflammation, thereby improving glucose metabolism abnormalities. Additionally, in a 24-week double-blind randomized control trial, researchers explored the effects of FMT combined with LSI in obese T2DM patients (110). By performing metagenomic sequencing of fecal samples, the study assessed the changes in microbial communities post-FMT and LSI, evaluating the impact on key health markers such as blood lipids and liver stiffness (110). Results showed that repeated FMT enhanced the engraftment and persistence of microbial communities in obese T2DM patients (110). FMT combined with LSI, led to beneficial microbial changes and improved blood lipids and liver stiffness. In a parallel study, a double-blind, randomized, placebo-controlled clinical trial assessed the dynamics of phage-microbiome interactions post-sterile fecal filtrate transplantation in patients with metabolic syndrome (111). Advanced techniques such as whole-genome shotgun sequencing and phage VLP sequencing were used to analyze bacterial and viral components of the gut microbiome. The study provided a thorough analysis of virus-host connections and statistical assessments, such as richness, α-diversity, principal component analysis, and phage-bacteria interaction dynamics. This research offered important insights into the effectiveness and safety of fecal filtrate transplantation, revealing its impact on the gut microbiome and virome, providing a new perspective for the treatment of metabolic syndrome.

In exploring the association and treatment strategies for NAFLD and its connection with T2DM, a randomized control trial targeting NAFLD and prediabetic patients offered profound insights. Analyzing fecal samples collected pre- and post-intervention through 16S rRNA gene sequencing, the study identified 5421 amplicon sequence variants (112). The research found that the combination of aerobic exercise and dietary intervention was associated with a diversified and stable keystone taxon in the gut microbial community, facilitating the diversity and stability of key microbes. Furthermore, individual exercise or dietary interventions increased the network connectivity and overall robustness between microbial taxa, suggesting these LSI can enhance the interconnectedness and adaptability of gut microbial communities to environmental changes.

Overall, these studies emphasize the key role of the gut microbiome in the management of T2DM and glycolipid metabolic diseases. Integrating lifestyle, pharmacological interventions, and omics methods has revealed the complex interplay between the gut microbiome and host metabolic health, influenced by a combination of host genetics, lifestyle, and pharmacological treatments. Future research directions should focus on developing drugs targeting specific microbial communities, aiming to specifically modulate the composition and function of the gut microbiota, thereby improving the metabolic state of T2D patients.

## 6 Integrated application of non-omics biotechnological techniques in the study of glycolipid metabolism in type 2 diabetes mellitus

In the field of glycolipid metabolism research for T2DM, the latest studies have focused on innovative biotechnological interventions that go beyond traditional omics methods. For instance, Dorzagliatin, a dual-acting glucokinase activator, showed significant enhancement in patients’ insulin secretion and glucose sensitivity in a double-blind, randomized, crossover study involving GCK-MODY and T2D participants (113). The effect of Dorzagliatin on wild-type and mutant glucokinase activity was meticulously analyzed using NADP+ coupled assays and *in vitro* studies with glucose-6-phosphate dehydrogenase. This finding revealed how drugs acting directly on key enzymes of carbohydrate metabolism impact the overall glycolipid metabolic process, offering profound insights into enzyme kinetics and laying a foundation for understanding the drug’s impact on metabolic pathways. Furthermore, research on β2-agonists, such as clenbuterol, further demonstrated the potential role of drug interventions in improving insulin sensitivity (114). The effect of β2-agonists on peripheral glucose handling under insulin stimulation in healthy males was evaluated through muscle biopsy and GLUT4 translocation assessment, revealing the potential role these drugs play in promoting insulin sensitivity and influencing glycolipid metabolic processes. Subsequently, the study of Sotagliflozin and Ertugliflozin elevated glycolipid metabolism research to new heights. This research delved into the comprehensive impact of these two drugs on metabolism, intestines, and cardiovascular system in T2D patients (115). Clinical samples, including blood, urine, and feces, were analyzed to detect various biomarkers such as blood sugar, insulin, GLP-1, and GIP, as well as 24-hour urinary and fecal electrolytes and SCFAs. The study employed mixed meal tolerance tests, continuous glucose monitoring, and ultrasonography to assess the drug’s impact, revealing significant changes in metabolic parameters consistent with intestinal SGLT1 inhibition, particularly post-breakfast effects of sotagliflozin aligned with intestinal SGLT1 inhibition. Additionally, biochemical changes in urine and feces provided clues about how these two drugs impact patients’ metabolism. The research then shifted to explore the impact of dietary types on glycolipid metabolism. By comparing the effects of dietary palmitate and oleate on insulin sensitivity in human skeletal muscle, the study found that fatty acid types significantly influence insulin signaling and glucose metabolism, offering a new perspective on the role of dietary factors in glycolipid metabolism (116).

In the research of T2DM and its complications, a series of innovative studies from preclinical experiments to in-depth clinical analysis are advancing the field. Firstly, in the study of diabetic peripheral neuropathy, an innovative approach using human pluripotent stem cells derived Schwann cells demonstrated how to model the disease and discover new treatment pathways (117). Through this method, researchers successfully generated cells with primitive Schwann cells molecular characteristics capable of forming myelin *in vitro* and *in vivo*. The study established a diabetic peripheral neuropathy model, revealed the selective vulnerability of Schwann cells to hyperglycemia-induced glyotoxicity, and discovered that the antidepressant drug bupropion effectively combats this glyotoxicity through high-throughput drug screening. Further research indicated that treatment with bupropion under hyperglycemic conditions could prevent sensory dysfunction, Schwann cells death, and myelin damage. This study not only showcased the research strategy combining innovative biotechnologies with experimental pharmacology but also emphasized the importance of interdisciplinary approaches in addressing complex complications of diabetes, introducing new therapeutic strategies and drug targets in the field of T2DM glycolipid metabolism research. Additionally, a key study explored metabolic abnormalities of intestine-derived lipoproteins in T2DM patients under statin therapy (118). Using stable isotope tracing, the study tracked the dynamics of ApoB-48 and ApoB-100 lipoprotein particles in patients’ bodies. The findings revealed significant differences in postprandial ApoB-48 levels and its kinetics in the VLDL conversion process compared to healthy controls, highlighting its potential role in cardiovascular health (118). Furthermore, oral intake of Blautia wexlerae as a gut microbiota-based intervention showed significant effects in improving obesity and T2DM (119). This intervention initially stemmed from the analysis of tissue samples and cell lines from non-small cell lung cancer patients, focusing particularly on the expression of NANOG and OCT4. Subsequently, this finding was extended and validated through animal model studies, linking laboratory discoveries to preclinical intervention strategies. Following this, the RECOGITO trial explored the gender-specific impact of tadalafil on diabetes cardiodynamics in a 20-week double-blind placebo-controlled clinical trial. This trial not only demonstrated its efficacy in the clinical phase but also provided biological evidence of the treatment effect through in-depth analysis of patient biological samples. This included measuring serum biomarkers via Enzyme-Linked Immunosorbent Assay measuring tadalafil concentrations via ultra-high-performance liquid chromatography, and assessing microRNA via digital droplet Polymerase Chain Reaction. This comprehensive approach enabled researchers to more holistically assess the biological effects and potential mechanisms of the treatment. Notably, in another study, researchers conducted an in-depth analysis of visceral adipose tissue samples from obese patients and normal-weight control subjects (120). Using the patch-clamp technique, researchers measured the function of the SWELL1 channel in human adipocytes and analyzed the expression of the SWELL1 protein in adipose tissue using Western blot technology (120). The aim of this study was to explore the role of the SWELL1 channel in T2DM and its complications, particularly Non-Alcoholic Fatty Liver Disease. These studies collectively demonstrate the research strategy of combining advanced molecular biology techniques with experimental pharmacology, emphasizing the importance of interdisciplinary approaches in addressing complex complications of T2DM, and introducing new therapeutic strategies and drug targets in glycolipid metabolism research.

## 7 Translating multi-omics findings into clinical practice

Multi-omics approaches bridge the gap between complex biological data and practical clinical applications, thereby enhancing disease prediction, drug efficacy forecasting, and drug selection for T2DM in multiple aspects. Specifically, transcriptomic, proteomic, and metabolomic profiling illuminate variations in gene expression, beta-cell dysfunction markers, and metabolic disruptions. These insights enable clinicians to customize treatments to the unique biological profiles of each T2DM patient. A notable development in this field is the polygenic risk score, a method for evaluating the genetic predisposition to specific diseases (121). These scores effectively categorize patients into categories of higher or lower risk for conditions like cardiovascular disease (122). Moreover, significant initiatives such as the UK Biobank and the Million Veterans Project are gathering vast biological data and performing comprehensive omics assays on millions, promising to revolutionize our understanding of human diseases. These initiatives aim to create predictive models of disease risk, enhance early detection, and improve disease management strategies (123).

As an essential component of modern drug discovery, the role of multi-omics in drug target identification is becoming increasingly prominent (124). The comprehensive approach of integrating multi-omics data, including genomics, transcriptomics, proteomics, and metabolomics, has been proven to facilitate the discovery of new drug targets and the customization of treatment strategies. This integration helps identify which biological pathways are altered in a patient, allowing for more precise targeting of these pathways with drugs. In a study, the researchers identified Blautia as a potential bacterial modulator using multi-omics technology in cohort studies, and oral supplementation of Blautia in T2DM mice demonstrated inhibitory effects on lipid accumulation, weight gain, and blood glucose levels while modulating gut bacterial composition, highlighting the clinical significance of stool microenvironment stratification and Blautia supplementation for microbial precision treatment of metabolic diseases (125). This method not only aids in discovering new therapeutic targets but also enhances the ability to predict a patient’s response to existing medications, thereby optimizing treatment outcomes (126).

## 8 Exploring individual variability and personalized treatment strategies in T2DM

Personalized medicine is otherwise called precision medicine tries to find better prediction, prevention, and intervention for T2DM individuals (127). Breakthrough of technologies enables much greater improvements in the understanding of individual variations that may alter the T2DM outcome. Personalized medicine, which tailors therapies, disease prevention, and health maintenance to individual needs, leverages multi-omics—including genomics, transcriptomics, proteomics, and metabolomics—as a key tool to enhance outcomes and minimize adverse effects. Precision medicine in diabetes refers to the utility of multi-omic data of a patient with diabetes to provide the most effective diagnosis strategies and treatment plans. For example, a study involving the use of polygenic risk scores (PRS) demonstrated that individuals with high genetic risk for T2DM who adhered to personalized lifestyle interventions had a significantly reduced incidence of diabetes compared to those receiving standard care (25). In another example, patients with T2DM exhibiting specific proteomic signatures were treated with SGLT2 inhibitors, resulting in improved glycemic control and reduced cardiovascular risk. These cases show the potential of personalized medicine to transform T2DM management by leveraging multi-omics data to inform treatment decisions​​​​ (125).

Additionally, the shift from pharmacogenetics to pharmacogenomics, now covering fields such as proteomics and metabolomics, has expanded the application of genomics across numerous therapies—from proteins to irradiation—across various medical fields (128). We argue that combining pharmacogenomics and pharmacomicrobiomics will provide an important foundation for making major advances in personalized medicine (129). The rising number of FDA approvals for biomarker-driven personalized therapeutics highlights the growing influence of pharmacogenomics (130). Molecularly targeted cancer therapies exemplify this shift in drug discovery and clinical application, while integrated biomarker panels that blend genetic, personal, and environmental factors are increasingly enhanced by artificial intelligence, refining diagnostics and treatments. In conclusion, the integration of advanced omics technologies into clinical practice not only enhances personalized medicine but also sets new standards for managing diseases like T2DM, paving the way for more tailored treatment approaches by discovering novel markers of phenotypes and treatment effectiveness.

## 9 Integrating omics into diabetes patient care

Incorporating individual variability into T2DM treatment strategies through personalized medicine approaches holds significant potential for improving patient outcomes. By leveraging multi-omics technologies, clinicians can develop tailored treatment plans that address the unique genetic, epigenetic, proteomic, and microbiomic profiles of each patient. This comprehensive approach enhances the effectiveness of treatments and paves the way for innovative advancements in T2DM management. However, translating these multi-omics findings into routine clinical practice involves several critical steps and infrastructural developments. Ensuring the practical implementation of these insights is essential for advancing personalized medicine in T2DM.

Firstly, Clinical Decision Support Systems that incorporate multi-omics information can help clinicians tailor treatment plans to individual patients, improving therapeutic outcomes. For example, the integration of polygenic risk scores from GWAS into electronic health records (EHRs) enables clinicians to stratify patients based on genetic risk factors. Such systems have been piloted successfully, demonstrating improved patient management through personalized treatment plans (131, 132)​​​​. This approach aligns with the challenges identified at the 2016 ASCO Data Standards and Interoperability Summit, highlighting the necessity for systems capable of managing complex genomic data. Integrating these systems into clinical workflows is crucial to enhance precision in patient care (133). However, these systems require significant investments in technology and training, posing a barrier to widespread adoption.

Secondly, standardization and validation of multi-omics biomarkers are essential for their clinical application. The urinary proteomic classifier CKD273, for instance, has been standardized to predict disease progression, guiding early intervention strategies. Validation through large-scale clinical trials ensures the reliability and reproducibility of these biomarkers, making them suitable for routine use in clinical settings​​ (132). The importance of clinicians understanding and effectively utilizing genetic information is underscored by findings from a national survey, which revealed a direct correlation between genetic education and the rates of referral to genetic evaluation. This correlation highlights the critical role of comprehensive genetic training in enabling effective management through multi-omics insights (134). On the other hand, the lack of standardized protocols across different laboratories can lead to variability in results, underscoring the need for universal guidelines.

Further, effective implementation necessitates robust collaboration between researchers, clinicians, bioinformaticians, and healthcare providers. Interdisciplinary teams can facilitate the translation of complex omics data into clinical protocols. Initiatives like the Diabetes Research on Patient Stratification (DIRECT) consortium exemplify successful collaboration, bringing together expertise from various fields to translate genetic findings into clinical practice (135). Fostering such collaborations can accelerate the development of novel therapeutic strategies tailored to individual patient profiles.

Educating healthcare professionals on the utility and interpretation of multi-omics data is also vital (136). Training programs and workshops can equip clinicians with the necessary skills to utilize omics data in patient care. For instance, involving clinicians in the research process can bridge the gap between discovery and implementation, ensuring that findings are clinically relevant and applicable. However, continuous education is required to keep pace with the rapidly evolving field of omics technologies.

Moreover, several real-world applications underscore the feasibility of multi-omics integration in clinical practice. Transcriptomic data, for instance, has been used to identify gene expression changes in pancreatic islets, informing the development of targeted therapies for improving insulin secretion and β-cell function in T2DM patients (37). Similarly, microbiome analysis has led to personalized dietary interventions that improve glycemic control, demonstrating the direct clinical benefits of omics research (102).

Finally, the implementation of multi-omics in clinical practice must also address data privacy and ethical concerns, including informed consent and the use of genetic information, must be rigorously managed to maintain patient trust and compliance. Organizations often lack the social license to use personal health data beyond its initial intent. Although some technology companies have principles for the ethical use of AI, they often overlook the acceptable use of the data itself. A community compact, as envisioned by a patient research network, aims to create a socially and ethically responsible data contract (137). A multistakeholder working group emphasizes commitments to data stewardship, governance, and accountability, including continuous learning, respecting individual choice, informed consent, people-first governance, open communication, accountable conduct, and inclusivity (138). Balancing privacy concerns with data accessibility will enable a more effective and ethical implementation of multi-omics in clinical practice.

In summary, the successful translation of multi-omics findings into clinical practice requires a multifaceted approach, encompassing technological integration, standardization, interdisciplinary collaboration, education, and ethical governance. By addressing these elements, we can harness the full potential of multi-omics to enhance T2DM management and patient outcomes.

## 10 Summary and outlook

### 10.1 Summary

In this review, we have comprehensively explored the pivotal role of biomarkers in the pathophysiology of T2DM and demonstrated the significant contributions of multi-omics approaches to understanding and treating T2DM. Initially, genomic studies provided new insights into the genetic diversity of T2DM, particularly significant genetic variations identified in GWAS, elucidating the role of genetic factors in disease progression and highlighting the need for multi-ancestry research. We then focused on the role of DNA methylation, underscoring the importance of epigenetic mechanisms in T2DM pathology, especially the potential of MRS in predicting different T2DM subtypes and their complication risks. Additionally, findings in transcriptomics, particularly through single-cell RNA sequencing, offered new perspectives in understanding the cellular mechanisms of T2DM. In proteomics, our unbiased approach to exploring key protein networks, such as the discovery of TBC1D4 protein, provided a deeper understanding of the molecular basis of insulin sensitivity. The application of metabolomics techniques revealed key signs of early metabolic dysregulation in T2DM, especially in lipidomics, offering new directions for early diagnosis and intervention. Lastly, microbiomics research emphasized the complex interactions between gut microbiota and host metabolism, revealing the potential role of gut microbiota in the risk of prediabetes in T2DM. Molecular biology techniques have opened new windows for in-depth exploration of the molecular mechanisms of T2DM, particularly the molecular pathways of β-cell dysfunction. These studies not only revealed specific mechanisms of β-cell damage but also pointed out new treatment strategies, such as inducing β-cell regeneration through the disruption of the DREAM complex.

In our interventional studies of T2DM clinical samples, the effects of drug and LSI were evident in significant changes in biomarkers and metabolic profiles. Notably, changes in biomarkers resulting from drug interventions reflected the profound impact of disease management, such as improvements in glycemic control, lipid metabolism, and inflammatory response. Through detailed analysis of biomarkers in blood and tissue samples, we were able to track and quantify these changes. Certain drugs, like metformin and insulin sensitizers, exerted significant regulatory effects on blood glucose levels by impacting insulin signaling pathways and lipid metabolism routes, clearly mapped at the genomic, transcriptomic, and proteomic levels. Moreover, metabolomic analysis revealed changes in levels of metabolites like branched-chain amino acids and fatty acids post-drug intervention, associated with insulin sensitivity and metabolic health. LSI, such as dietary changes and increased physical activity, also showed significant effects on T2DM biomarkers and metabolic profiles in our research. These interventions triggered changes reflected in the adjustment of gene expression patterns, alterations in metabolite concentrations, and reductions in inflammation markers. For instance, patients adopting high-fiber or Mediterranean diets exhibited significant improvements in fatty acid metabolism and energy metabolism pathways in blood and tissue samples. These findings are crucial for enhancing insulin sensitivity and glycemic control, demonstrating the immense potential of early lifestyle changes and nutritional adjustments in reducing the global burden of T2DM. Particularly in high-risk groups, the effectiveness of preventative measures is vital for reducing disease incidence and complications. In the field of non-omics technologies, our review highlights the application of advanced biotechnologies such as human pluripotent stem cell-derived Schwann cells, and their importance in modeling disease complications and discovering new therapeutic pathways. For example, the application of bupropion in combating insulin sensitivity disorders and diabetic nephropathy, as well as the role of statins in regulating intestine-derived lipoprotein metabolism. Additionally, research on fecal microbiota transplantation (FMT) showed the potential for improving glycemic control by altering gut microbiota, highlighting the profound impact of LSI on microbiome composition.

These studies underscore the importance of utilizing multi-omics approaches for a comprehensive assessment of T2DM treatment effects. Through such a holistic analysis, we can better understand how drug and LSI affect the biological basis of T2DM and grasp the nuances of disease management. This multidimensional approach reveals the complexity of treatment effect assessment, indicating that reliance on a single biomarker or pathway may not comprehensively reflect therapeutic efficacy. In summary, our research emphasizes the importance of adopting multi-omics approaches in T2DM treatment research, which not only aids in precise diagnosis and treatment but also contributes to providing more personalized and comprehensive treatment strategies for patients.

### 10.2 Discussion on the importance of research methodology

In this review, we have explored the critical role of biomarkers in the pathophysiology of T2DM, showcasing the substantial contributions of multi-omics and various non-omics biotechnological techniques. As the future of personalized and precision medicine evolves, treatment strategies for T2DM are shifting from traditional ‘one-size-fits-all’ approaches to more individualized interventions. Central to this shift is the integration and analysis of data from omics and various non-omics techniques, offering insights into the genetic and molecular basis of the disease and guiding treatment approaches for specific patient groups. Omics techniques such as genomics, proteomics, and metabolomics provide tools for an in-depth understanding of the molecular mechanisms of glycolipid metabolic diseases, enabling identification of biomarkers for disease progression and potential therapeutic targets. For instance, analyzing patients’ genomic data can reveal how specific genetic variations influence the risk and efficacy of diabetes treatment, laying a foundation for precision medicine. Proteomics and metabolomics analyses shed light on biomolecular changes in disease states, offering vital information for developing new therapeutic methods. Consequently, multi-omics analyses combining genomics, transcriptomics, proteomics, and metabolomics can elucidate the multilayered pathological mechanisms of T2DM. This integrated approach helps understand how various biological levels interact and impact disease progression and treatment response. Enhancing the application of these omics methods and expanding their impact necessitates the use of high-throughput screening technologies, artificial intelligence, and machine learning algorithms. These technologies enable rapid analysis of vast amounts of gene, protein, and metabolite data, revealing subtle differences between patient groups and predicting individual responses to specific treatments. This not only enhances the personalization and precision of treatment strategies but also accelerates the translation from data to clinical application.

Beyond omics technologies, non-omics biotechnologies play a significant role in T2DM research. Examples include the application of microfluidic techniques in studying pancreatic islet cell function and the in-depth exploration of insulin signaling abnormalities through cell culture and molecular biology techniques. Additionally, *in vitro* disease models and organ-on-a-chip technologies complement T2DM research, providing valuable platforms for drug screening and mechanistic studies by more accurately simulating the microenvironment of human organs. Their integration with omics and non-omics techniques offers powerful tools for a more comprehensive understanding of T2DM and its treatment.

Thus, by combining clinical research findings with in-depth laboratory studies, we can better translate research outcomes into clinical applications. For example, investigating genetic and molecular changes in patient samples and then validating these findings in a laboratory setting can expedite the development and optimization of new treatment methods. This integration of methodologies and innovative applications heralds a new era in T2DM treatment and management. The findings and discussions of this review highlight the key role of interdisciplinary approaches in understanding and treating T2DM, offering new perspectives for comprehensive disease management. Through a multidimensional research approach that combines omics techniques with fundamental experimental methods, we can not only better understand the pathophysiological mechanisms of T2DM but also lay the groundwork for developing more effective treatment strategies. This comprehensive research approach paves the way for future diabetes research and clinical practice.

### 10.3 Limitations

In reviewing the research and treatment methods for T2DM, several key limitations must be acknowledged, highlighting current research challenges and guiding future directions. Firstly, the representativeness of samples and limitations in data sources may impact the universal applicability of conclusions. Reliance on data from specific populations or regions could lead to limited applicability to other groups or areas. Additionally, translating laboratory findings into clinical practice remains a significant challenge. Although our review proposes a range of promising treatment strategies, their clinical application requires validation through further clinical trials and empirical studies. Finally, our review may not encompass all areas related to T2DM, such as treatment strategies for specific populations or long-term drug side effects, areas for further exploration in future research. These limitations underscore the necessity of future research, not only in deepening our understanding of the complexity of T2DM but also in optimizing treatment strategies to more comprehensively address this challenging disease.

### 10.4 Potential of future precision medicine

Discussing clinical research and therapeutic mechanisms for T2DM underscores the importance of an integrated cross-omics approach, particularly in identifying new regulators of metabolic pathways, biomarkers, and novel drug mechanisms related to the condition. Omics technologies have revealed metabolic and lipid changes during the progression of T2DM, providing a deeper understanding of its pathophysiology. However, current studies have not yet reached a consensus on identifying core dysregulated metabolic pathways and potential biomarkers. Future research should focus on standardizing experimental procedures, using validation cohorts, and considering the openness of research data to generate more replicable and robust outcomes.

Applying various omics techniques and molecular biology can help us understand the metabolic dysregulation and treatment opportunities for T2DM more comprehensively. As these technologies advance, the future management of glycolipid metabolic diseases will increasingly emphasize individualized treatment strategies. Drug development will also increasingly depend on individuals’ genetic and metabolic characteristics to enhance therapeutic effects and minimize side effects. Exploring multi-target drugs is crucial for developing new treatment strategies, particularly those that synergistically target multiple biological processes in T2DM. These drugs may offer therapeutic effects on various aspects of the condition, such as improving insulin sensitivity, regulating blood sugar levels, and reducing complications. Management of individuals with T2DM involves not just customizing drugs and treatment plans based on their genetic backgrounds and biomarkers but also integrating lifestyle interventions, such as diet, exercise, and behavioral changes, to optimize treatment outcomes. These technologies also provide opportunities for early diagnosis and prevention of glycolipid metabolic diseases, enabling interventions before clinical symptoms manifest by identifying early biomarkers and risk factors. This comprehensive and personalized management model not only improves treatment efficacy but also enhances the quality of life for patients and reduces overall healthcare system costs. Therefore, the application of omics and non-omics technologies in optimizing chronic disease management is a key factor in advancing the treatment and prevention of glycolipid metabolic diseases.

Clinical trial validation and improvement of existing treatment methods, as well as the development of new treatment strategies, will be crucial in advancing personalized medicine. Furthermore, integrating multi-omics data will aid in comprehensively understanding the metabolic dysregulation and treatment opportunities in T2DM. Through these integrated and innovative approaches, future research in personalized medicine and precision treatment for T2DM will not only provide more effective treatment options but also significantly improve the quality of life and health outcomes for patients. Ultimately, our research should focus on utilizing these technologies and methods to discover biomarkers effective in early detection of T2DM and indicators that can predict disease progression and treatment response. Through long-term prospective studies and comprehensive analysis of clinical samples, we can better understand the complexity of T2DM, provide more precise treatment strategies for individuals, and bring new advancements in the management and treatment of T2DM.
